# WHEN LIFE DOESN'T GO AS PLANNED: UNMET EXPECTATIONS ABOUT RETIREMENT TIMING AND SUBSEQUENT DEPRESSIVE SYMPTOMS

**DOI:** 10.1093/geroni/igz038.584

**Published:** 2019-11-08

**Authors:** Leah R Abrams, Neil Mehta

**Affiliations:** University of Michigan School of Public Health, Ann Arbor, Michigan, United States

## Abstract

The 2008 Great Recession affected American’s retirement timing, but it remains unclear how unfulfilled expectations about retirement timing influence psychological well-being. This study examines how unmet expectations about working at age 62 relate to subsequent depressive symptoms, with special attention to sociodemographic differences in unmet expectations and their consequences. We use longitudinal data from 10,557 adults ages 51+ in the Health and Retirement Study (1994-2014). Mean expected probability of working full time at age 62 (ranging 0-100) was 40.5 (SD=54.65). We created quartiles: no chance (0 probability, 35% of sample), unlikely (1-30, 16%), unsure (33-80, 28%), and very likely (85-100, 21%). Expected probability and the association between expectations and reality were significantly lower for racial minorities compared to whites, low education compared to high, and pre-baby boomers compared to baby boomers. Those who were working at age 62 but expected to be retired did not experience elevated depressive symptoms compared to those who correctly expected to be working. In contrast, those who were unexpectedly not working experienced significantly higher depressive symptoms compared to those who correctly expected to be retired (Unsure: IRR=1.16 p=0.024, Very likely: IRR=1.19, p=0.010). This association was slightly attenuated after adjusting for declines in functioning, suggesting partial, but not complete, mediation by health status. The association was consistent by race, education, and birth cohort, but was larger in men than women. Taken together, our findings indicate that unexpected continued employment does not harm psychological well-being, but earlier than expected retirement may result in higher depressive symptoms.

